# Pathological Anatomy

**Published:** 1836-01-01

**Authors:** 


### Medico-Chirurgical Review,

### No. XLVII.

**[No. 7 of a Decennial Series.]**

OCTOBER 1, 1835, TO JANUARY 1, 1836.

### WORKS ON PATHOLOGICAL ANATOMY.

1. OUTLINES OF HUMAN PATHOLOGY. By *Herbert Mayo*, F.R.S. &c. Professor of Anatomy, Physiology, and Pathological Anatomy in King's College, London; Surgeon to the Middlesex Hospital. Octavo, pp. 264. Part I. price 8s. London, 1835.

2. ILLUSTRATIONS OF THE ELEMENTARY FORMS OF DISEASE. By *Robert Carswell*, M.D. Professor of Pathological Anatomy in the University of London, &c. Fasciculus Eighth—Pus. London, 1835.

THE first work upon the list is a fresh acquaintance, introduced by an old friend, Mr. Mayo. The reputation of that gentleman must ensure his protégé a favourable reception. The second work is a continuation of the Illustrations of Pathological Anatomy, by that able and zealous pathologist, Dr. Carswell. Without further preface, we shall proceed to consider each seriatim.

The first chapter of Mr. Mayo's work is consecrated, of course, to the pathology of the bones, that punctum saliens of all anatomists, natural and morbid. The pathological phenomena displayed by them are arranged by Mr. Mayo under the heads of—reparation—hypertrophy—atrophy—inflammation—abscess—necrosis—caries—malignant growths—hydatids.

The first section is occupied with the description of the process and phenomena of reparation of bone. The description is possessed of the useful merits of clearness and conciseness; but, as it merely states what elementary works have already made familiar, we may pass it over without further notice.

### HYPERTROPHY OF BONE.

Hypertrophy, Mr. Mayo defines to be the abnormal increase of a part, without inflammation or change of structure. This hypertrophy of bone may affect a part only, or the whole—the partial hypertrophy being termed an exostosis. Mr. Mayo is of opinion that, under the head of hypertrophy of bone, affections of very different kinds are classed; some nearly connected with the most salutary principles in the economy; others constituting obstinate but curable disorders; others, again, being diseases of a troublesome or even malignant character. This imputation of vagueness of nomenclature relates, we suppose, chiefly to partial hypertrophy or exostosis. We were not aware of any great attention having hitherto been directed, or of any great confusion having yet attached to the notions of general hypertrophy of bone.

It is to exostosis that Mr. Mayo first proceeds; and, as a good account of this "partial hypertrophy" is really a desideratum, we shall notice his arrangement and his observations. He considers hypertrophy under nine heads.

1. He observes that it is a principle in the animal economy, that exercising a part serves to strengthen it. This principle is displayed in the increase of muscles, bones, and other textures, under exertion of the part they supply. In Mr. Cheshire's apparatus for weakness of the spine, the chin and the ossa ilii are compressed, or rather they have to sustain much pressure. The former bone, in patients in whom the apparatus is used, generally enlarges, and throws out a bony swelling at the part where the chin-strap tells. This is hardly, strictly speaking, an example of exostosis, a disease; for, on discontinuing the application of the instrument, the swelling disappears.

2. In infancy and childhood, the progress of growth is rapid. At those periods of life, the bones sometimes grow out irregularly and excessively. The clavicle is a bone most subject to this variety of exostosis. The disease requires no treatment, for, in the course of a year or two, either the rest of the bone grows up to the enlarged surface, or the superfluous portion is absorbed.

3. Exostoses are most frequent when the body has just attained its full growth. Yet the later periods of life are not free from them. A patient sixty years of age applied to Mr. Mayo, with an exostosis of the first rib, which threw forward the subclavian artery, so as to give the appearance of a subclavian aneurism. He had not observed the swelling till some months before he applied for advice.

"The common situations of exostoses are the upper part of the humerus and of the tibia, and the lower part of the femur. Their shape varies with the kind of bone upon which they grow. In the long bones, they are generally narrow, of greater height than thickness; sometimes largest at their base; sometimes the reverse. In the flat bones they are usually broad, with no great elevation; sometimes they form flat discs, resting by short narrow pedicles upon bones of this class. In the round bones, they are rounded and nodular. To these laws, however, there are many exceptions.

Exostoses present in their structure all the varieties which healthy bones exhibit. Sometimes their grain is dense and compact; this is especially the case with those that grow from the cranial bones, as upon the temporal or frontal. In other instances, they have a crust of the consistence of, but of less thickness than that of the bone on which they grow, joined to a cancellous structure of the ordinary density. They appear, in some instances, as if formed by the development of cancelli in a part of the cortex: in other words, the cortex which covers them, and that on which they rest, are together of the thickness of the cortex of the sound bone adjacent.

Exostoses have not more sensibility than healthy bone: but they are susceptible of inflammation, and then become the seat of pain; and they often occasion pain by their pressure upon more sensible parts. When in the neighbourhood of important organs, they are liable to disturb their functions. Fixed pains in the head, and epilepsy, have been produced by the growth of exostoses from the inner table of the cranium.

The causes of exostoses cannot generally be traced: it is certain, however, that they are occasionally mechanical; such as pressure or a blow.

Exostoses sometimes spontaneously disappear. Their absorption is promoted by counter-irritants, mercury, iodine. When removed by an operation, they generally do not recur. A young woman was a patient in the Middlesex Hospital, under Mr. Cartwright, with a humoral exostosis, which, after several remedies had been tried, was sawn off. In a year another exostosis grew, nearly in the same place; but on a rubefacient plaister being applied over it, an abscess formed, and the new bone was absorbed. Exostoses sometimes perish by necrosis." 13.

4. Sometimes the tumor which forms an exostosis is not, in the first instance, bony. In a child at the age of six months, a soft tumor was observed to grow from the front of the alveolar process of the upper jaw. Sir Astley Cooper removed the tumor; but it grew again, and gradually became hard. The subject of the disease grew up a fine young woman, but for the deformity occasioned by the tumor, which projected two inches and a half, and pressed the upper lip against the nose. Mr. Mayo removed the morbid growth, which proved to be an exostosis of cancellated, but extremely hard bone. It is three years and a half since the operation, and the tumor has not returned.

5. Exostoses, of a texture dense and compact, like ivory, occasionally grow from the bones of the orbit and the cylindrical bones, and attain considerable dimensions. The disease may become serious, the eye being protruded from the socket and sight lost, or the limb being rendered an useless incumbrance. The disposition to the formation of ossific tumors may prevail throughout the system, and display itself in other textures besides the bones. Number 533 (dry pathological specimens in the Hunterian Museum) is a preparation of ossification of the lungs, from a patient who died of pulmonary disease, supervening after amputation of the leg for ivory exostosis. Mr. Stanley possesses a specimen, in which it exists in combination with medullary sarcoma.

6. There is a preparation, in the King's College Museum, of a tibia and fibula greatly enlarged, but not appearing to have been inflamed. The structure of the bones is healthy. The person of whom Mr. Mayo purchased this specimen, assured him that the bones were from a Barbadoes leg.

7. A patient died in the Middlesex Hospital, who had been afflicted with epilepsy. The convolutions of the brain were remarkably flattened. On examining the cranium, the inner table of the frontal, parietal, and occipital bones, was found hypertrophied; all the arterial grooves were of unusual depth. The bones were not particularly vascular. The cranial bones, at least those of the vault, are well known to be not unfrequently much thickened in cases of idiotcy, and even mania. We lately examined the body of a child, who had long been epileptic. The frontal, parietal, and occipital bones were thickened to a great degree.

8. " A sort of hypertrophy of bone is the extension of ossification into the ligaments and muscles. In a preparation, in the King's College Museum, the femur is fixed immovably in the acetabulum by ossification of the anterior part of the capsular ligament, and of part of the iliacus internus. Mr. Langstaff, in his rich pathological collection, has a beautiful specimen of ossification, which appears to have spread from the femur, and involves the vastus internus, the structure of which is converted into bone, but preserves externally its fibrous character." 15.

Of this kind, we suppose, is that species of exostosis so frequent at the point of insertion of strong muscles—for instance, of the deltoid into the humerus, and of the ligamentum patellæ in the tibia. Mr. Mayo makes no mention of the frequency of exostosis in these particular situations, yet still it should be known that they *are* frequent.

The alliance between the ossification of ligament, muscle, tendon, and exostosis is well shewn, says Mr. Mayo, in the remarkable skeleton called Mr. Jeff's in the Hunterian Museum. The printed catalogue thus describes it, and, as many of our readers may never see either that or the original, we are tempted to insert the description here.

"It is the skeleton of a man thirty-nine years of age, and is remarkable for the production of ossific growths from many parts, of various dimensions and extent; some forming exostoses merely, whilst others pass from one part of the skeleton to another, and have thus produced anchylosis, or immobility of most of the members. The exostoses may be observed in the os frontis, mastoid process, and occiput; and in other parts of the skeleton where muscles are attached, as near the angle of the jaw, where the masseter is inserted; at the extremities of the spines of the vertebræ, at the coronoid processes of the ulnæ, in the femur, at the part where the glutæus maximus is implanted.

The second and more extensive kind of ossifications have in general followed the course of the larger muscles, and may be seen, on the right side, in the situation of the deltoid, joining the clavicle and acromion of the scapula to the humerus, in the situation of the supra-spinatus, and passing from the inferior angle of the scapula to the humerus, in the situation of the teres major and latissimus dorsi. On the back, more extensive ossifications of the muscles appear, which affix the scapula on both sides to the sacrum and ilium, and to the spines of the lumbar and dorsal vertebræ. On the left scapula, the ossification of the teres major has not extended quite to the humerus; but the dorsum presents a singular process or ossification, with smooth sides, and a flattened overhanging margin, like an auxiliary or second spine.

From the pelvis, ossifications extend from the sacrum and ilium in the direction of the glutæus magnus; and from the tuber ischii and os pubis in the course of the biceps flexor and triceps adductor muscles; these extend to the right femur. Ossifications of the tendinous and ligamentous parts are still more common, producing anchylosis of the vertebræ, of the left elbow-joint, of the tibia and fibula to each other on both sides of the ancle-joint, and general consolidation of the bones of the tarsi." 16.

9. A general disposition to hypertrophy of bone is found, in those who have suffered from the opposite defect in childhood. In most specimens of recovery from rachitic curvature of the spine, the edges of the vertebræ are enlarged, and encroach upon the intervening ligaments. In the bones of the extremities, strong ridges are in like manner thrown up along their concave aspects. Mr. Stanley has pointed out that there is method in this abnormal growth, and that the superabundant bone is placed exactly where the curvatures of the bones render them mechanically weaker.

Such are Mr. Mayo's remarks upon hypertrophy of bone—such his divisions and general account of exostoses. We think there are several varieties of this species of morbid growth, which he has omitted to include in his category, and certainly there are several circumstances connected with them to which he has not drawn attention. Yet, with these defects, the classification of the facts is still worthy the attention of the reader. The subject of the next section is—

### Atrophy of Bone.

Atrophy is defined to be degeneration of growth, deficiency in the size and weight of a part, and commonly of one or more of its usual constituents in particular. Atrophy in bone is attended with deficiency of bone earth, and with more or less alteration of the gelatin of bone and of the medullary membrane. Mr. Mayo enumerates seven varieties of atrophy of the osseous structure.

1. Disuse weakens parts, as use strengthens them. In a limb long unused from disease, the bones, as well as the soft parts, become atrophied. A young woman had her leg amputated for disease of the tibia, which had existed for eleven years, and had prevented all use of the limb. Where not swollen by disease, it wasted. The head of the tibia was a thin shell of bone, and, instead of its proper close cancellous structure, a few fine threads only of bone were drawn across it. Among these the marrow lay in large masses, semi-fluid, supported by the medullary membrane alone.

2. In childhood, and especially in female children, the round bones are liable to a form of atrophy which corrects itself with advancing years. The weakness involves the ligamentous structures. It shows itself at the ancles, at the knees, but principally in the back: the spine becomes curved from weakness, inclining to one side at the loins, but being simultaneously bent at the upper part in the opposite direction, so that the trunk may preserve the perpendicular. This complaint is atrophy from arrest or retardation of structural growth. The weight and size of the child increase faster than the hardness and strength of its bones and ligaments. Sometimes the spine is twisted, sometimes incurvated forward as well as laterally.

3. In old age the bones become weaker and more brittle, the cortex being thinner, and, in the cancelli, the due quantity of bone earth being deficient. This is what some authors have termed the interstitial absorption of bone. It is the cause of the frequency of fracture of the neck of the thigh-bone in old persons, and of the sinking of the head and shortening of the neck of the femur at the same age. In the museum of the school at Kinnerton Street are the thigh-bones of a person advanced in life. In each, the bone is apparently quite healthy, except that it is lighter than it should be, whilst in both the head is half an inch below the level of the summit of the trochanter major, the axis of the cervix horizontal, and the cervix itself about half its usual length.

4. "Sometimes in middle life, without any assignable cause, the bones are unusually brittle. A stout-looking man was a patient in the Middlesex Hospital, with some trifling ailment. He was cutting a slice of bread, when the humerus broke. The bone united as readily as another bone: and this is observed in general of atrophied bones, except in extreme cases, or where a malignant growth exists in addition in the bone; or where the system is scorbutic, or tainted with lues or mercury." 18.

Even in cases where malignant disease does exist, and bones are fractured in consequence, union sometimes occurs without difficulty. Sir Benjamin

Brodie is in the habit of mentioning an instance of this in the case of a lady who laboured under cancer of the breast, and in whom cancer involved the bones.

5. In persons, says our author, affected with carcinoma of the breast, the ribs are commonly found atrophied, so that they admit of being cut with a knife. The cortex is thin; and the osseous plates and threads of the cancelli are thinner and fewer than natural. The cells contain a reddish-brown and slightly gelatinous fluid. The bones of the extremities are likewise often unusually brittle. Sometimes, but very rarely, carcinomatous tumors are found in these brittle bones.

In a patient who died in St. George's Hospital, of some affection, we forget what, unconnected altogether with malignant disease, the ribs, and indeed the majority of the bones, were altered in this manner. The humerus could be easily cut through with a knife, and it seemed extraordinary that the long bones had not broken in the ordinary motions of the body.

6. No definite line can be drawn between common atrophy of the bones of children and rickets, every intermediate degree of weakness being observed. In rickets the physical changes are:—the cortex is thinner than usual, and more porous; and the bony cancelli have disappeared. The bones in the worst cases again have a consistence approaching that of common cartilage: but their texture is areolated. The cells are in some parts large, and contain a brownish gelatinous substance.

7. A certain degree of atrophy of bone occasions brittleness—a greater, produces flexibility with increased brittleness. This disease attacks adults, but is of very rare occurrence—it is named mollities ossium. There have been very contradictory descriptions of this malady, and, being uncommon, the ideas entertained respecting it are very vague. Mr. Wilson's description appears to us to be that of malignant disease of bone, and not mollities ossium. We extract Mr. Mayo's remarks upon the subject, because they embody an account of two cases, more completely tallying with a consistent notion of the real characters of mollities ossium, than the majority of such cases do.

"In the museum of the College of Surgeons, there is a specimen of an adult humerus, of which the cortex is as thin as a wafer, and the interior looks as if filled with tallow: towards the lower part, for about two inches above the condyles, the bone appears to be a vascular sac of membrane. Mr. Hunter considered this to be mollities ossium. In the museum of the London Hospital are specimens from the body of a woman, who died at the age of seventy-two, which nearly resemble that above referred to. This woman had been confined to her bed, for four years, with paralysis of the lower extremities. The hip and knee-joints had been for a considerable time permanently fixed: her appetite was always good. A month before her death, on making a slight exertion, the right thigh-bone broke: and, shortly after, the right arm. Mr. Hamilton, with great liberality, has presented to the King's College museum a preparation of one of the bones from this body; and the following is his account of the general *post mortem* appearances.

'The lungs were healthy; the heart rather large and soft; the abdominal viscera and mesenteric glands healthy. There was considerable calcareous deposit in the lumbar and iliac glands. In the cranium, when the dura mater was opened, three or four ounces of fluid escaped, and there were some small tubercles attached to its under surface. The substance of the liver was natural. The articulations were in a healthy state. The periosteum covering the bones generally appeared natural; but over the trochanters it was entirely detached. The bones of the cranium and pelvis could be cut with a knife; while the ribs and vertebræ were but slightly affected, and scarcely less firm than usual. The bones of the lower extremities were far more extensively diseased than those of the upper. The thigh bones consisted of a mere shell of bone, filled with a fatty substance: the fractured extremities of the femur and humerus had a slight ligamentous connexion.'" 20.

In the fourth volume of the Medico-Chirurgical Transactions, Dr. Bostock describes an analysis, by himself, of "two of the dorsal vertebræ of an adult female, whose bones were discovered, after death, to be unusually soft and flexible," the result, as it was supposed, of mollities ossium. The composition of the bones was as follows:—

| | |
|---|---|
| Cartilage................ | 57.25 |
| Jelly and oil ............ | 22.5 |
| Phosphate of lime ........ | 13.6 |
| Sulphate of lime.......... | 4.7 |
| Carbonate of lime ........ | 1.13 |
| Phosphate of magnesia .... | .82 |
| | 100.00 |

### Simple Inflammation of Bone.

We perceive nothing to detain us in this chapter. Indeed we should observe that there is what we may term, in Irish language, a vein of incompleteness throughout these sections. Individual facts are thrown together, but though they are each of course a part, they do not together form a whole. Certainly the present section offers any thing but a perfect history of inflammation of bone. The only passage that appears to require notice refers to inflammation of the ribs.

"I have seen," says Mr. Mayo, "in living persons, two instances of simple inflammatory enlargement of the ribs: one occurred in a young lady, whose general health was not otherwise considerably impaired: the result of the case is unknown to me. The other had the following history. A young gentleman, through hard study and neglect of exercise, became dyspeptic, with pain in the head, and inability to collect his thoughts. After a time, three or four of his ribs on each side enlarged, and were painful. He gave up his studies, and went to the south of Europe, where he recovered. A year or two afterwards, he became a medical student in London. In a little time, his former indisposition returned; and he became again dyspeptic, with hypochondriasis, and painful swellings of the ribs. Upon going into the country, he recovered." 25.

### Abscess in Bone.

That abscess should occur in a bone as a consequence of inflammation is not à priori unlikely. Yet prior to some observations published by Sir Benjamin Brodie in the Medico-Chirurgical Transactions, no distinct account of the affection had appeared. To those observations we directed full attention very lately, and we do not think it necessary to go into the subject again.

### Necrosis.

Mr. Mayo furnishes a fair account of the history and characters of necrosis. But the chief interest in the section is connected with the practice of removing the sequestrum before Nature would, unassisted, be enabled to effect its discharge. The reasons for the interference of art are obvious—the earlier termination of the disease, and, consequently, the diminution of the sufferings and of the injury to the patient's health. Sir Benjamin Brodie, in his valuable clinical lectures on necrosis, has drawn the attention of surgeons to the advantages of trephining the new bone in this disease, and removing through the openings made by the instrument part of or all the sequestrum. We have frequently seen Sir B. Brodie perform this operation with success, success that exhibits in a pleasing point of view the improvements effected by modern surgery in the treatment of this common, severe, and therefore interesting disease.

The following case detailed by Mr. Mayo will enable those of our readers who are unacquainted with the clinical lectures of Sir B. Brodie, and with his practice in this complaint, to understand the latter.

"James Mills, ætat. eighteen, in April 1834, received a severe blow on the left tibia, through a chest falling against it. The bruised part was rubbed with vinegar. He walked about as usual during the next ten days. The leg then swelled and became painful, when leeches and fomentations were used. At the expiration of a week, a surgeon opened the swelling, and a considerable quantity of matter was discharged; afterwards other openings were made, and for eight or ten weeks the limb was poulticed. The patient then began again to walk about, the leg continuing to discharge, but without much pain. Towards the beginning of January, however, the pain became again aggravated. He was now unable to walk, the leg being easy in the horizontal posture only. The upper part of the tibia appeared enlarged to three times its proper bulk. At either end of the enlarged part, and in the middle, were sinuses. A probe introduced into these passed through an opening in an outer shell of bone, and came in contact with a sequestrum within. Towards the end of March, the sequestrum was so loosened, as to yield when pressed upon by the probe. An incision was therefore made through the integuments covering the shell of bone, and the thickened skin dissected far enough on either side to give room for the application of a middle-sized trephine over the centre of the enlarged part. A circle of bone having been removed, the sequestrum was exposed, which I cut in half with bone forceps. The trephine hole was then enlarged sufficiently to allow the parts of the sequestrum to be drawn out." 32.

Mr. Mayo notices the modes of necrosis in different bones under different circumstances. In the flat bones, as is well known, necrosis is usually observed in the form of exfoliation. Sometimes a large extent of a flat bone dies, and is separated wholly. Such a case occurred to Mr. Mayo. A person sixty years of age fell from a cart, and grazing his head against the wheel, denuded the parietal bone. Towards the end of the eighth month, nearly the whole of that bone came away : the surface exposed was that of the granulating dura mater. No attempt at reparation by bone had taken place.

The lower jaw can hardly be deemed a flat bone. In that we have ourselves seen evident restoration by new bone. Partial restoration is said to have occurred after necrosis of the scapula.

Exfoliation of the articulating extremity of a bone, or of a part of it, sometimes occurs into a joint. In such cases the joint is generally destroyed; they are not very uncommon. Necrosis in these instances is usually conjoined with caries. Necrosis of the bones of the wrist and tarsus, when it occurs, as it is sure to involve a joint, is equally destructive.

Necrosis of the os calcis, however, is an exception; the bone is of a size to allow of disease in it being partial. The necrosed part may exfoliate, and the bone recover.

### Caries.

Caries in bone is strictly analogous to ulceration in soft parts, and, like it, is preceded by inflammatory action. Mr. Mayo divides caries into four varieties. 1. Simple caries. 2. Syphilitic caries. 3. Strumous caries. 4. Malignant caries.

1. *Simple Caries.* Mr. Mayo gives in illustration of this form, a suppositious case. It has been observed, he says, that the bones of the leg are particularly disposed to inflammation. In persons of a constitution originally sound, hard labour, exposure to weather, bad and insufficient nourishment, co-operating with a dependent circulation, as they frequently produce ulcers of the leg, so do they occasionally produce caries of the bones of the leg. The tibia becomes inflamed, and then, instead of recovering or forming a circumscribed abscess, slowly enlarges: the cortex, already more spongy than natural, is next eaten through at one or more points: the cancellous structure within in the mean time is softened: and its cells are occupied with a thick brown fluid in place of marrow. The disease is often coupled with death of the bone, part of the carious bone undergoing necrosis. The external character of the limb is the same in necrosis and caries. The bone appears enlarged, and one or more sinuses open from it through the skin at points which are red, and soft, and sunken. He then gives several isolated cases. Of this form of describing a disease we may simply say, that it either does too little or too much. Either the cases shew all the known varieties, or they do not. If they do exhibit every form, the mode of doing so is diffuse and roundabout; if they do not shew all the forms that exist, they are of course imperfect, and are still objectionable from diffuseness and circumlocution. In an elementary work of this sort, the statements should be plain, the general principles clear and comprehensive; when cases are detailed, it should be for the purpose of special illustration, or for shewing a rare fact that no general principle contains. Mr. Mayo adopts the contrary method. His sections may be said to be chiefly made up of a set of cases strung together, sometimes without apparent order. This may do very well for a reader thoroughly acquainted with the subject, but certain we are that it never will do, for those who must form the majority of readers —pupils and men imperfectly versed in pathology.

2. *Syphilitic Caries.* Mr. Mayo's remarks upon this head are meagre. He makes no mention of the agency of mercury, in producing nodes and caries of bone, yet nine times out of ten "syphilitic caries" is as much mercurial as syphilitic. Syphilis, indeed, has credit in this as in other instances, for more than it deserves. Mr. Mayo makes a remark which attracted our attention, because it is not founded on very accurate observation. He observes:—" the co-existence of ulcerated fauces, and squamous eruption or other diseases of the skin, generally leaves no doubt as to the nature of the caries." Now, in point of fact, squamous eruption is not an usual companion of caries—the eruption is in the vast majority of cases of the ulcerated character, attended with scabs.

3. *Strumous Caries.* " This in many cases, bears a close resemblance to syphilitic caries: in other instances it is difficult to draw the line between it and simple caries. In a third class of cases, certain peculiar and characteristic features are strongly marked.

A young lady, whom I have attended, has suffered from inflammation of the periosteum of the tibiæ in oval patches resembling nodes. Matter has slowly formed, and the bone has been exposed in a carious state; small exfoliations have then taken place, and the wounds have healed. Three swellings of this description have formed in succession. Her appearance gives evidence of the strumous diathesis.

A gentleman thirty-seven years of age, in whom the strumous habit is clearly marked, consulted me for caries of the palate. The alveoli of the upper jaw were likewise carious. He was liable to inflammation of the tonsils, attended with ulcers. The complaint, however, was not of syphilitic origin. The sister of this gentleman has had caries and partial necrosis of the turbinated bones.

A young man, twenty-five years of age, became my patient for caries of the head and face. A small exfoliation had taken place from the os malæ of the left side, and from the right superciliary ridge. The integuments of the right side of the forehead were swollen, puffy, and tender: there was discharge from the nose, and part of one turbinated bone was necrosed. No ground existed for supposing that the disease was of syphilitic origin.

There is not, that I am acquainted with, any essential difference in the appearance of carious bones in this form of scrofula, and in the parallel cases dependent upon lues. Less pain, less periosteal inflammation, and a smaller extent of surface attacked, the absence of other symptoms, and the general physical appearance of the patient, afford a strong presumption of the scrofulous origin of the disease.

The bones of the face are peculiarly susceptible of scrofulous inflammation. The character of the complaint is often clearly established by the formation of extensive scrofulous abscesses upon the temple or upon the cheek, or at the angle of the jaw; and by the chronic enlargement of the lymphatic glands of the neck.

Of the bones of the head, the frontal and the temporal are the only ones which I have known involved in this form of strumous caries.

The sternum is often affected with strumous caries, characterised, as in the face, by the formation of extensive scrofulous abscesses. The seat of the suppuration is the anterior mediastinal cavity. The bone may require to be perforated with the trephine, to give vent to the matter." 42.

We have seen extensive strumous caries in the parietal bone of the head of a young woman. But certainly the most usual situations of strumous caries are the ends of long bones, and the cancellated structure of the round and mixed varieties of bones, the tarsus, the carpus, and the vertebræ. We have seen many instances of strumous nodes upon the tibia, especially in young females. They seem to begin with inflammation of the periosteum, followed by suppuration between it and the bone, and ultimately caries of the latter.

Mr. Mayo makes a remark, which though just, may deceive the inexperienced reader. He observes that some will include under the head of simple caries cases which he shall describe as scrofulous, and that it is certain that fewer diseases are now considered scrofulous than formerly. The latter observation is strictly true. Yet pathologists are becoming, perhaps, too prone to resolve all actions into one, and to banish all ideas which wander from their own abstract standard of unity. The cultivation of pathology has tended to this evil, slight indeed in comparison with its important services, and slighter because it leads after a time to its own correction. M. Andral, one of the most philosophical pathologists of the day, has confessed the error to which pathology has given birth, and in his excellent work has done justice to the correctness of that empiric observation, which of itself discovered that all processes in the human body are not similar, and that one mode of action, call it what we will, will neither explain nor produce all the phenomena presented to our observation.

We apprehend, to return to the specific remark of Mr. Mayo, that although the term strumous was formerly greatly abused, yet that many affections must still be admitted to arise from a condition analogous to what we know as a scrofulous diathesis. It is impossible to explain these peculiar effects, by supposing them to arise from simple inflammation. Some modifying agency there evidently is, and if we do not choose to call that scrofulous, we shall only be forced to call it something else.

It is difficult to deny, and few do deny the correctness of general principles like this. It is their application which occasions the disputes. In order to diminish doubt and difficulty, precision of ideas and terms is necessary. We should agree to some general definition of scrofula, discriminate its characters, and ascertain the pathological condition of bone, which accompanies them. Such is the mode of evidence which would satisfy the philosophical reasoner, and a lesser degree should be rejected. Let us see how Mr. Mayo manages the question. He brings forward this case as an instance of scrofulous caries.

"I amputated the leg of a patient, twenty-five years of age, five months after a compound fracture of the tibia and fibula. The bones had shown a disposition to unite, but the general inflammatory swelling of the limb which supervened upon the accident, never subsided. Large collections of matter formed in the calf of the leg, and about the ankle. These were opened in succession, and the patient as many times rallied from the hectic fever and exhaustion which they produced. At last there formed above the knee-joint an extensive abscess; which was opened; when a profuse discharge of matter took place, and afterwards of blood and matter alternately. It was now that the limb was amputated, but too late to save the patient. In the knee and ankle joints the cartilages were found partly absorbed, and what remained reduced to a thin shell. The articular aspect of the bone, which had become exposed, was highly inflamed. The narrowed and thin shells of cartilage which were left, were found to tear very readily from the bone: when torn off, the detached surfaces were found to be covered with bony particles, showing that the separation must have been effected by rupturing the inflamed surface of the bone. On further examination, the whole articular aspect of the condyles was found to be highly inflamed and softened, for the depth of one to two lines. The bone beyond was perfectly healthy." 44.

The above *might* be an instance of scrofulous caries of the bone, no doubt; but we see no satisfactory evidence that it was so. No mention is made of the presence of the ordinary marks of a strumous habit—it was not previously shewn by dissections, nor indeed by arguments, that the pathological state discovered, is a state confined to or extremely frequent in scrofulous cases—and, in short, we are told, and we may believe it if we choose, or dispute it if we feel inclined, that this was an instance of strumous caries, and the declaration that it is so is at once the assertion and the proof.

The characters that really denote scrofulous inflammation in bone, are shewn in the work of Sir Benjamin Brodie, by the process of investigation we have mentioned. They are—the deposition in the cancelli of tuberculous matter, an unorganized substance of a yellow colour and of the consistence of curd. We need say no more on this point, as we lately presented so very full a notice of Sir B. Brodie's work. We therefore proceed to the last kind of caries described by Mr. Mayo, the ulceration of the bones in lupus, and the ulceration of the jaw in cancer. But his observations offer nothing to detain us.

### MALIGNANT GROWTHS OF BONE.

Mr. Mayo describes six varieties of malignant tumors of bone existing either separately or in combination. They are, malignant exostosis, osteosarcoma, medullary sarcoma, fibrous tumor, cyst-like tumor, melanosis.

Of malignant exostosis no further is said than was mentioned in the section upon exostosis.

*Osteo Sarcoma.* We extract Mr. Mayo's description of this, as it combines perspicuity with brevity.

"It consists," he says, "in a growth of substance, nearly resembling epiphytic cartilage in texture, originating either upon the surface or in the cancelli of bone. The form of the tumour is commonly more or less spherical: it may attain so great a volume as to be nearly a foot in diameter.

When an osteo-sarcoma is small, the surface displayed by a section is tolerably uniform, or differs from the most transparent cartilage only in exhibiting minute oblong or irregular cavities. When an osteo-sarcoma is larger, cavities of a considerable size are found in it, which contain a reddish fluid. In parts the texture grates when cut, and contains phosphate of lime. The phosphate of lime is distributed so as to form a kind of skeleton of light bony plates disposed in a manner that looks like a crystallization. The growth of such a tumour is commonly rapid. When it begins in the interior of bone, the disease is attended with pain: when it forms on the outer surface, there is commonly no pain at all. An osteo-sarcoma has to the touch the firmness and elasticity of cartilage. This disease is ordinarily met with in the bones of the extremities, and in the lower and upper jaw. The cranial bones and the vertebræ are less frequently, if ever, attacked by it. The disease does not, that I know of, invade any other texture than bone: it bears, however, some external resemblance to gelatiniform cancer of other parts. It has not much malignity, so that when all the bone involved in it, with part of the adjacent sound bone, is removed by amputation, the complaint seldom re-appears either in the part, or in another bone. If the part is not amputated, the skin over the tumour sloughs or ulcerates, the tumour is exposed, and a discharge, sanious or ichorous, takes place from it, under which the patient gradually sinks." 51.

We may make two observations upon the foregoing description. We doubt whether osteo-sarcoma can be separated so clearly and so satisfactorily from medullary and other alterations of bone, as Mr. Mayo seems disposed to think. A boy had the thigh amputated for a growth possessing all the characters attributed here to true osteo-sarcoma, which grew from the internal condyle of the femur. The wound made by the amputation was not quite healed, when symptoms resembling those of phthisis supervened. The boy died, and tubercles of medullary sarcoma were found to exist in the lungs. We believe it is acknowledged that morbid growths from bone resembling in their origin osteo-sarcoma, may finally be converted into true malignant degenerations. Here then we see two reasons why osteo-sarcoma can scarcely be altogether separated from other malignant tumors of bone, and probably the amount of our present knowledge is little more than this—that tumors possessed of these particular characters are less generally followed by the evidences of constitutional contamination, than other malignant growths, and that though they sometimes become confounded with the latter, they are sometimes found distinct from them.

Mr. Mayo relates an interesting case of osteo-sarcomatous growth from the tibia. The tumor was dissected off the bone. A fungous tumor sprang from the cicatrix. Amputation was then performed above the knee. The stump healed, but the cicatrix again ulcerated, and the disease re-appeared at the end of the femur. Mr. Mayo proposed amputation at the hip-joint, which the patient very judiciously declined, and, the tumor slowly increasing, he died. Mr. Mayo remarks that—the practical deduction from the preceding case is that, in osteo-sarcoma, it is a degree safer to amputate at a joint, than to risk, in a constitution disposed to this action, the exciting it in another bone by the saw. We do not clearly perceive the force of this conclusion. The case proves certainly the ill success of amputation; but it does not, nor do we see that it possibly can prove, that no re-appearance of the malady would have occurred, if another operation had been practised.

Mr. Mayo makes a remark, the propriety of which we are disposed to doubt. "When new formations (he adds) appear in bone with the local characters of malignant disease, they have not the same rootedness in the system, as when they originate in the soft parts; so that the removal of a bone attacked by malignant disease is not in general followed by a return of the complaint. For example, in one who has suffered amputation of the breast for medullary sarcoma, the disease is sure to recur; but when the leg is amputated for the same disease originating in the tibia, the chances are greatly in favour of the patient's permanent escape." We almost fear that Mr. Mayo overrates the chances of escape after operations for malignant growths from bone. Did we trust our own observation and experience, we should say that the disease appears in other parts, in a very large proportion of cases. We could call to our memory, at the present moment, too many instances of failure after amputation of a limb.

3. *Medullary Sarcoma.* On this we have only two remarks to make. 1. Mr. Mayo declares that medullary sarcoma of bone generally, if not always, arises in the cancellous structure. That it does not always do so, we are certain. In two cases of medullary sarcoma of the femur, the disease clearly originated in the superficies of the bone, or between it and the periosteum; and, in an instance of medullary sarcoma of the ilium, we noticed the same thing. At the same time it must be owned, that the cancelli form the most frequent nidus for this morbid growth.

4. *Fibrous Sarcoma.* There is a form of malignant disease of bone, of which the texture is firm, white, and fibrous. Its origin, Mr. Mayo believes, and its place, to be exclusively periosteal.

This disease is met with on the tibia. If simply removed from the bone it grows again : the limb must be amputated.

The same growth is liable to form upon the cranial aspect of the dura mater, to push its way through the bone by absorption, and to project great masses of sarcomatous growth upon the head and face.

5. *Cyst-like Tumor.* In the heads of the bones of the extremities, and in the lower jaw, a disease, which has the general characters of malignant growth, is found, when examined after death or amputation, to consist in a great cyst, or series of cysts, containing gelatinous liquid.

6. *Melanosis* in bone is rare.

" The diseases which have been enumerated are as often met with combined as separately.

Mr. Stanley possesses a specimen of ivory exostosis, combined with medullary sarcoma.

Mr. Stanley gave to the King's College museum a section of a tumour upon the femur, which he had amputated. It consists, at one part, of medullary sarcoma ; at another of fibrous sarcoma. The medullary sarcoma appeared to have originated in the cancelli of the bone, and had caused absorption of the cortex, which became extenuated, and the femur broke. The fracture is surrounded by a large soft tumour ; part of this tumour is medullary, part consists of a firm, white, opaque substance, not cartilaginous.

In a leg amputated at the Middlesex Hospital for malignant disease, the cancelli of the tibia contained brain-like substance, or true medullary sarcoma. The crust of the bone was thin and brittle. The muscles of the back of the leg were externally healthy ; but near the bone, in place of their proper texture, there was substituted a firm white substance, corresponding to Mr. Abernethy's description of mammary sarcoma. The fore part of the leg had a remarkable tenseness and brawny hardness : the skin was red and thickened. The former appearances were produced by a very singular lobulated subcutaneous texture, to which Mr. Abernethy's term of pancreatic sarcoma was strikingly applicable. This part of the disease appeared to me a conversion of the adipose tissue into a malignant growth.

There is an excellent case related in the 120th Number of the Edinburgh Medical and Surgical Journal, illustrating at once the connexion between osteosarcoma and medullary sarcoma of bone, and a remarkable feature, which is occasionally present in the latter disease—a pulsation, namely, as if the tumour were aneurismal. The pulsation is probably communicated from the contiguous arterial trunk." 55.

These remarks, taken with what we have incidentally observed, will serve, we fear, to shew that, however we may artificially arrange the various morbid growths from bone, much uncertainty still exists with respect to the real nature of each, and the actual amount of relationship of all. It is certain that they are more or less convertible, and more or less combined, and, in the present condition of our knowledge, we can hope for no more than a general approximation to the truth. The practical deduction is, we fear, unsatisfactory—that too many of these morbid growths are liable to return after an operation, in the site of the wound or in other parts, and that the knife should be employed early, if at all.

"True scirrhus in bone I suppose to be of rare occurrence. I have mentioned that there exists, in the King's College museum, a specimen which looks like a scirrhous tumour, which was found in the medullary cavity of the femur of a person labouring under cancer; and there are in London several other preparations of a similar description. But I am not acquainted with an instance of an undoubted scirrhous *enlargement* of bone.

Mr. Sweatman has a remarkable specimen of scirrhous periosteum. A woman, about seventy years of age, was a patient in the cancer ward of the Middlesex Hospital, for carcinoma of the breast. About a month before she died, one eye was observed to protrude; and three days before her death she became suddenly comatose. Upon examining the skull, the dura mater and pericranium, and orbital periosteum, for a considerable extent on the affected side of the head, were found to be thickened and hard: the dura mater was, at one part, a third of an inch in thickness; the arachnoid adhered to it, and partook in the same thickening. The bone is not diseased, but is something more vascular than usual.

In the museum of the College of Surgeons there are several specimens of thickening of the pericranium and dura mater. No. 607 is a section of the right temporal and parietal bones of a young woman, twenty-five years of age. A tumour projects externally, about half an inch above the surface of the parietal bone; and there is a similar tumour situated exactly opposite, on the inside of the skull. These tumours appear, in the preparation in spirits, not unlike that just described, except that the part towards the skull appears opaker than the rest. The opposite section, however, is preserved dried; by which means the opaker part is shown to consist of short bony threads, in close apposition, which have no continuity with the cranial bones, on which they rest, but must have formed within the tumour. The tumour I suppose to have been malignant periosteal growth. The intervening portion of the cranium is sound, but unusually vascular." 57.

### Hydatids in Bone.

All writers who have noticed this affection of bone, have transcribed the case, or an epitome of the case, published by Mr. Keate in the tenth volume of the Medico-Chirurgical Transactions. In that case the hydatids were found in the os frontis, just over the left eye-brow. The case has been so frequently quoted, and must now be so familiar, being contained more than once in the pages of this Journal, that we may safely pass it by.

This terminates the Pathology of the Bones. The second Chapter is devoted to that of the Articulations. On casting back a glance at the pages we have quitted, we perceive some interesting facts, and what is professed, an outline of a systematic arrangement. Yet much, of course, is matter devoid of novelty, and even what has some claims to novelty, is, probably, from the very nature of the subject, in some degree obscure and doubtful.

### The Joints.

Mr. Mayo considers first the diseases of the Synarthroses, or Immoveable

Articulations, and, secondly, those of the Diarthroses, or Moveable Articulations.

*Synarthroses* exist between the bodies of the vertebræ, and between the pelvic bones. Mr. Mayo first enumerates, which we need not do, the component textures of these articulations, and then their properties and their alterations.

" Fibro-cartilage, when torn, is susceptible of reparation. In fracture of a vertebra, the adjacent fibro-cartilage is generally ruptured; if the patient lives, it unites just as bone unites. The texture of the cartilage of a rib is perhaps too dissimilar to that of intervertebral substance, to be used in illustrating the properties of the latter; but I may take the present opportunity of mentioning some experiments made by myself upon reunion of these parts. The cartilage of a rib was divided in several animals, which were killed at different periods afterwards. I found the initiatory stages of reparation which had been set on foot to be exactly similar to those in bone. The cellular membrane surrounding the divided part was first consolidated into a firm capsule, which contained the cut ends of the cartilage. This consolidation was produced by infiltration with lymph; an exudation of the same substance formed a medium of direct union between the ends of the divided cartilage. The reparatory capsule gradually became converted into a texture resembling cartilage. As, in the reparation of bone, the callus changes into cartilage, and then ossifies, subsequently to which direct union of the broken ends by bone takes place, so, in the restoration of a costal cartilage, the exterior thickening becomes cartilaginous, while the direct union of the divided ends is still by lymph alone." 63.

Fibro-cartilages are susceptible of absorption, through pressure made upon them, but the quantum of absorption is less in them than in bone. In aneurism of the descending aorta, the intervertebral fibro-cartilages are found to be only superficially absorbed, when the bodies of the vertebræ are already deeply excavated. Mr. Mayo thinks that the intervertebral cartilages are partially absorbed also in cases of lateral curvature of the spine.

" Fibro-cartilages generally, it may be presumed, are susceptible of inflammation. The only instance, however, in which I have found this demonstrable, occurred in the semi-lunar cartilages of a knee-joint. Ulceration of the cartilages covering the bones had taken place, with high inflammation of the adjacent surface of the bones, and of the capsular synovial membrane. The semilunar cartilages (the knee having been injected after amputation) were red with the vermilion, swollen, and softer than natural; and when divided, showed, upon the surface of the section, extremities of cut vessels." 63.

Fibro-cartilages may undoubtedly ulcerate, become the seat of suppuration, and slough. These morbid changes are seen in the intervertebral substances.

Mr. Mayo classes the diseases of the vertebral column under two heads—atrophy and inflammation. The first is exhibited in the lateral curvature of young persons—the second in ordinary spinal disease. To this subject, however, we need advert no further, in consequence of the full manner in which we treated of it, in our review of the work of Sir Benjamin Brodie. The only other observations on the subject of diseases of the synarthroses, which deserve particular attention are contained in the following quotation.

" Disease of the pelvic joints is of unfrequent occurrence. At the time of labour, however, an affection occasionally takes place in these joints, which is sometimes distinctly inflammatory, while at other times it bears the character of simple absorption of the fibro-cartilage. I witnessed an instance in which, after labour, an abscess formed behind the symphysis pubis, which was attended by a sense of weakness and giving of the pubic joint, which lasted several weeks, but gradually went away. Cases of this description are the most common. But sometimes the sacro-iliac joints are principally affected: there is no suppuration, but extreme weakness at these joints, which lasts many months. The patient is obliged for a long period to keep the recumbent posture; and then, and afterwards while recovering strength, derives remarkable comfort from bandages round the pelvis.

The following case, which was under my care, exemplifies commencing disease in the sacro-iliac synchondrosis, brought on by external violence.

A gentleman was riding in Hyde Park, when his horse reared, and fell backwards, bearing him to the ground. He was lifted up by those around, when he found himself capable of walking, with assistance. I saw him a short time after the accident. The only part bruised was the integument covering the back of the sacrum, and more to the right side than the left. There was no fracture that I could ascertain, nothing but the bruise; and, as it afterwards appeared, a strain of the right sacro-iliac joint. The patient could bear the ilium to be pressed in any direction, and could, as I have mentioned, both stand and walk. In the evening considerable pain came on: he was cupped upon the hip, and experienced relief. The following day the cupping was repeated. After a month, during which this patient had kept his room, and the pain had nearly left him, he went, for change of air, to Richmond; when a child accidentally touching his foot, as he lay on a sofa, he drew up the limb suddenly. Upon this he experienced a sensation, which he described to be like displacement of the bones at the right sacro-iliac joint; and he fancied he recollected, that, at the time of the accident, he had felt a similar sensation; but certainly neither then, nor at this time, did pressure upon the ilium, in a direction to strain the sacro-iliac joint, bring on this sensation, or cause any thing like sensible motion of the joint. The pain now became gradually very severe, and extended down the limb, in the course of the sciatic nerve.

It was a year and a half from the occurrence of the accident before this patient had recovered. In this period many remedies were tried; those which were most beneficial were, strict observance of rest, and the application of caustic issues over the joint. When by these means the pain had been entirely subdued, cold sea-bathing rapidly restored his strength." 70.

We are disposed to agree with Mr. Mayo that slight inflammatory action, must be looked on as the ordinary and operative agent in the production of most of the alterations of the synarthrodial joints. No doubt the inflammation is of a low description, but the evidence and probability are in favour of its existence.

*Diarthroses,* or moveable articulations, and their affections, are next treated of by Mr. Mayo. The affections of the bones that enter into their composition he enumerates thus:—restorative action after fracture—atrophy—eburnation, or the solidifying into a texture like ivory—inflammation, with its numerous consequences—and deposite of scrofulous matter in the cancelli. The affections of cartilage are—reparation—softening—ulceration—inflammation—and gouty concretion. We may notice Mr. Mayo's account of the varieties of softening and of ulceration of which cartilage is susceptible.

" Cartilage is susceptible of two forms of softening; one may be considered true atrophy. It is often met with on the cartilage of the patella in persons a little advanced in years. The cartilage is softened, and seems split into soft, thick villi. The change is accompanied both by partial absorption of the cartilage, and by a growth of the ends of the isolated villi in delicate shreddy productions, to which the synovial membrane probably contributes. This condition of cartilage deserves to be viewed, not as disease, but as natural degeneration of tissue.

The second form of softening is of rare occurrence; in it the cartilage becomes semi-transparent and gelatinous. In a case of severe inflammatory disease of the knee-joint, with caries of the articular surfaces of the bones, and inflammation of the synovial membrane, I found near the crucial ligaments, for the extent a sixpence would cover, the synovial aspect of the cartilage on the inner condyloid cavity of the tibia softened and semi-transparent for two-thirds of its thickness.

Cartilage is susceptible of three varieties of ulcerative disease.

In the first, the cartilage disappears very rapidly, the absorption beginning upon the synovial aspect, leaving a surface perfectly healthy and smooth, either of cartilage or of bone. This change supervenes with great rapidity after compound dislocations, in which the wound does not heal, and the cavity of the joint remains exposed.

In the second, the process of absorption is slower, and produces an irregularly excavated and ulcerated surface on the synovial aspect. This condition of cartilage is a very common element in chronic articular disease. I believe it to be generally, if not always, preceded by synovial inflammation.

In the third, the process of absorption commences on the aspect of the cartilage towards the bone. I believe that this kind is essentially dependent upon inflammation of the articular aspect of the bone. The absorption takes place with two effects: one, the diminution of the thickness of the cartilage; the other, its detachment from the bone." 73.

The changes of synovial membrane and of ligament offer nothing to detain us.

Mr. Mayo having disposed of the individual morbid alterations of these individual tissues, proceeds to view the latter in their natural form of combination, as articulations—and the former in their natural combinations also, as groups of phenomena constituting the diseases actually presented to the observation of the surgeon. The heads under which he describes the affections of joints are these:—1. Injury and reparation. 2. Inflammation of ligaments. 3. Heightened sensibility of the synovial membrane, cartilages, and ligaments. 4. Inflammation of the synovial membrane with increased secretion of synovia, from local causes. 5. Inflammation of the synovial membrane with increased secretion of synovia, produced by causes of general or specific action on the constitution. 6. Inflammation and ulceration of the synovial membrane. 7. Inflammation of the synovial membrane without effusion, attended by rapid disappearance or absorption of the articular cartilages. 8. Inflammation of synovial membrane with chronic ulcerated excavation of the articular cartilage beginning on its synovial aspect. 9. Inflammation of synovial membrane with caries of the articular aspect of the bones. 10. Extra-articular inflammation, producing ulceration of cartilage, and caries. 11. Strumous caries of the ends of bones, producing articular ulceration. 12. Growths from the synovial membrane.

The phenomena attending injuries of joints and their reparation are first considered by Mr. Mayo. He shews that, after an injury, the wound may unite by the adhesive inflammation—or, that inflammation of the synovial membrane, followed by suppuration, ulceration of the cartilage, and so forth, may ensue. Sometimes the wound unites by the first intention, but, a copious secretion of synovia into the articulation ensuing, it gives way, and permits the escape of the fluid. Cases of this kind may be found in various works, and Mr. Mayo adds one to the list. Perhaps the most instructive is contained in the work of Sir B. Brodie. It is that of a gentleman from the knee-joint of whom he removed a fungous tumor. Mr. Mayo presents a concise account of the difficulties that obstruct the reduction of a dislocation, and interfere with the retention of the bones in situ when reduced. Yet these may be passed by.

Mr. Mayo relates some cases of inflammation of the ligamentous textures.

"*Case.* A gentleman struck his knee, and neglected it. Some months afterwards I saw him, when he laboured under the following symptoms. He had been now for many weeks confined to his bed or his sofa; the least exertion was followed by heat and pain in the knee. The joint was in a slight degree larger than the sound knee; but it contained no fluid; and pressure of the articular surfaces against each other produced no pain. It caused no pain to rest his weight upon the extended knee; but when he stood on the other leg, and allowed the diseased knee to hang or swing, he felt uneasiness in it, which was greatly increased by twisting the knee. When he remained perfectly still, he would experience no uneasy feeling in the joint for several days together; and then, without any visible cause, the joint became a little heated, and the skin slightly reddened. The same effects followed any deviation from the strictest rest." 80.

We have ourselves seen several cases in which there could be no doubt from the nature of the accident and of the symptoms, that the ligaments were the principal seat of inflammation. Those symptoms, we should say, may almost admit of resolution into two—pain on pressure of the ligament involved—and pain on the application of any thing which disturbs its state of rest. If the leading sign of affection of the cartilages of an articulation is pain when they are compressed, the leading sign of inflammation of a ligament is pain when it is stretched. But the production of suffering on the forcible elongation of the textures of a joint, is not certain evidence of affection of its ligamentous textures only, as affections of the synovial membrane, give rise to the same symptom. It must, therefore, be a combination of positive signs of implication of the ligaments, and negative signs in respect to the synovial membrane, that can constitute satisfactory proof of the affection of the former.

Mr. Mayo next adverts to an affection of the joints, which appears to be of what is called a neuralgic character. He says that the symptoms are limited to pain increased by motion and exquisite sensibility of the articular surfaces to pressure. He relates three cases, the two first for the purpose of displaying those symptoms, the third with the view of exhibiting, so far as that can be exhibited, its pathology. The latter case is the only one to which we think it necessary to direct attention.

*Case.* H. A. ætat. twenty-two, the catamenia regular, having suffered during four years pain in the knee-joint, which, although sometimes greatly mitigated, never entirely left her, at length, when every remedy that could be thought of had been tried, and the pain had much increased, underwent amputation of the leg. The symptoms had been pain and increased sensibility, and nothing more; the joint, with the exception of slight œdema arising perhaps from the local remedies, had not swollen, nor had there been any mechanical impediment to motion. On examining the amputated knee, which had been previously injected, the capsular synovial membrane was found of a bright red. The synovial membrane covering a small part of the semilunar cartilages was likewise very vascular. At the upper part of the patella, the same appearance was seen: towards the lower part, the synovial membrane, for the extent of five lines by two, was not only red with injected vessels, but considerably thickened. Soon after the stump healed, it was accidentally struck. To this cause, probably without reason, the patient attributed a return of pain exactly similar to that which she experienced before the amputation of the leg. When the pain had gone on several months increasing in severity, the patient, anxious at any expense of immediate suffering to get well, submitted to another operation. The pain and tenderness were seated in the last three inches of the stump, not more upon one aspect than another, although most acute as it seemed in the part of the cicatrix covering the bone. The extremity of the stump was therefore amputated, a second portion of bone sawn off, an additional portion of the sciatic nerve taken off in the operation, and the bone and nerve buried in a full bed of relaxed muscle and integument. On examining the part removed, the sciatic and the saphenus nerves were found to terminate in large white cartilaginous bulbs, behind but not adhering to the cicatrix. On the stump healing, the pain recurred, and, after some months of its continuance, the sciatic nerve was divided under the edge of the glutæus muscle. When the wound healed the sufferings of the patient were as great as before.

Unfortunately, this is not a solitary case, nor an unparalleled example of the want of success attending the performance of surgical operations for the relief of the symptoms and the sufferings that it displays. Whether the surgeon is justified in resorting to the knife in such cases, is a difficult question to determine. Sometimes it has brought relief, more often it has not. The patient is not unfrequently so anxious to catch at any straw, that he anticipates the surgeon, and may almost be said to demand an operation. As a general rule, such operations should not be countenanced, for they inflict present misery and unnecessary risk, and when unfortunate, as too commonly they are, in their results, they bring discredit on surgery and its professors.

When we say that these cases are neuralgic, we go very far indeed towards confessing that we do not exactly know what they are. It is what our Hibernian friends would denominate a negative affirmation. There is no great structural alteration discoverable, and for practical purposes a neuralgic malady of this description may be generally said to be functional.

But occasionally our pathology assumes a more decisive character. As in the instance of other disorders of the nervous system, for example epilepsy, we are sometimes gratified by what appears to be a tangible cause for the phenomena that were presented. Mr. Mayo refers to a specimen in the museum of St. Bartholomew's Hospital. The patient had such symptoms as were present in the case described already. After years of suffering, the knee was amputated, and no appearance of disease was discovered in the joint. Upon her death, which happened two or three years after the amputation, the spine was examined, and the posterior, or sentient surface of the chord was found studded with little plates of cartilaginous or bony deposit. Our ignorance is every now and then relieved, and our hopes are excited, by the discovery of such an Oasis as this in the desert pathology of the nervous system. Yet for once that we find a spot upon which we may satisfactorily repose, we are a thousand times compelled to abandon the idle search in despair.

We may pass over Mr. Mayo's description of inflammation of the synovial membrane—of rheumatism affecting it—and of gonorrhœal inflammation of the joints. But we may pause at "a class of cases of rare occurrence," first described by himself in the eleventh volume of the Medico-Chirurgical Transactions. With these our readers are probably not so familiar as they necessarily must be with the others.

"The disease consists in acute inflammation of the synovial membrane, with rapid absorption of the articular cartilage, attended, not with effusion into the joint, but with considerable œdema or suppuration external to it. The attack begins with sudden pain in a joint, and swelling round it, not attended with much symptomatic fever; and it frequently, in twenty-four to forty-eight hours, entirely leaves the joint first attacked, and settles in another. The pain continues for several weeks with hardly abated severity, then goes away, and the joint is found to be anchylosed. Leeches repeatedly applied, and superficial caustic issues, have appeared to me of manifest utility in lessening the pain." 90.

Mr. Mayo relates five cases in illustration of this description. We must candidly own that we think both the cases and description imperfect, and we do not feel quite sure that more than one affection are not comprised in this picture of an individual. We will, however, present one of our author's cases before we indulge in any further observations.

*Case.* A young woman, twenty-seven years of age, six months after labour, was seized with pain and swelling of the wrist. The next day, the right knee was attacked in the same manner. The following day, the pain and swelling left the joints first affected, and attacked and settled in the left knee. Six weeks after the commencement of her illness, Mr. Mayo saw the patient. The thigh and leg were swollen with serum effused in the cellular membrane; the joint contained no fluid; the pain in the knee was very acute and constant, and increased by the least motion; the patient lay on her back, with the knee straightened. The application of leeches to the part gave no relief; cold applications and fomentations were equally inefficient; repeated blistering appeared, at one time, to be of service; small doses of calomel and opium were administered frequently, during a few successive days, with no advantage. At length the pain seemed to abate spontaneously, the swollen state of the limb gradually subsided, the patient recovered the appearance of health, but the joint was wholly immovable.

To this case we think we should add another which is very short, and which is intended to illustrate the pathology of the complaint.

*Case.* A young woman was admitted into the Middlesex Hospital in the Summer of 1829, four or five weeks after her confinement, in a state of great emaciation, with a large abscess below the fascia of the thigh, extending from the knee to more than half way to the groin. The abscess did not communicate with the joint, which contained no fluid, but was contracted. The tibia was drawn backwards upon the condyles of the femur. In six weeks, this patient sank and died. Upon examining the knee-joint, the cartilage of the condyles of the femur was found to be entirely absorbed towards the edge, the bone exposed being healthy. The cartilage covering the condyles at other parts was uniformly attenuated, but smooth, and strongly adherent to the bone, which was healthy.

We seldom find very graphic descriptions very accurate. Something is and must be sacrificed for effect. We felt some misgiving on reading Mr. Mayo's description of the complaint. Acute inflammation of the synovial membrane, with rapid absorption of the articular cartilage, attended with no effusion in the joint, but with œdema or suppuration on its exterior, lasting almost unabated for weeks, then going away, and leaving the joint anchylosed, seem clear and curious enough in print, but not quite consistent with what one sees in practice. The first case detailed by Mr. Mayo affords some slight clue to this mystical pathology. That case has all the characters of one of rheumatic inflammation, and of nothing more. First one joint was affected, then another, and finally the malady settled in one knee, with the ordinary symptoms of ulceration of the cartilage. The disease as often happens in rheumatic affections of the articulations, terminated favourably in anchylosis.

In this, there is nothing to excite our surprise; it is a case which is not unfrequent. But such a case, or many such, would be insufficient to establish Mr. Mayo's positive annunciation of a form of disease, in which acute inflammation of a joint does *not* occasion effusion into it, but, having lasted for a certain time, passes off, and the articulation becomes anchylosed. So far as we can see, the affection described by Mr. Mayo is generally of a rheumatic origin, that is, occurs under the circumstances which usually give rise to rheumatic inflammation. But it is not the joint alone which is affected; the cellular and fibrous textures external to it are involved in the disease. Sometimes they are more severely implicated than the joint itself —sometimes the affection of the latter is secondary, and produced by the extension of the inflammation from without. We have seen such cases, exactly tallying with the description offered by Mr. Mayo—and we have seen others, in which that description is obviously imperfect. In the former instances, the disease was not sufficiently intense to occasion suppuration in the joint—in the latter that occurred, and, instead of a favourable termination being the result, ulceration of the cartilages, suppuration within the joint and without it, and death, have been the course and consequences of the complaint. In short, we believe that Mr. Mayo's notice of this particular form of articular disease is too exclusive, and exceedingly imperfect —that he has selected for its subjects the slighter forms of the affection to which we have alluded, and probably other species of diseases of the joints also—and that a more extended observation and experience will lead him to correct the narrow picture he has drawn.

In order to illustrate our own remarks, we will briefly mention the particulars of a case or two which we have witnessed.

*Case.* A girl was admitted into St. George's Hospital, under the care of the late Mr. Rose. She was of a strumous habit. She had been exposed to cold a short time previously to her reception in the institution. One knee was swollen, but the swelling extended also up the thigh, and down the leg. There was great pain on pressure of the articulating surfaces together, or, indeed, on any motions of the joint, and there was also extreme suffering on pressure of any of the swollen part of the thigh or leg, but particularly of the former. Leeches, blisters, splints, and calomel and opium, were the means employed. The symptoms gradually subsided, and the patient recovered, with an anchylosed knee-joint.

*Case.* A young female, some weeks after parturition, was attacked with swelling of the lower part of the left thigh. In the course of ten days or a fortnight, she was admitted into St. George's Hospital. The lower two-thirds of the thigh were much swollen, pitted on pressure, and were extremely painful when touched or moved. There was also great pain in the knee-joint, which was rather swollen. Leeches, calomel and opium, &c. were employed, but the patient was seized with the symptoms of low fever, and, in six or seven weeks after her admission into the hospital, she died. On dissection of the limb, the swelling of which had greatly diminished, there was found suppuration under the vastus internus muscle, and an ulcerated aperture in the synovial capsule allowed this matter to communicate with the joint. In the latter, the cartilages were partially ulcerated.

*Case.* A washerwoman, after exposure to cold and wet, was suddenly attacked with swelling and violent pain in the thigh and knee. In a very few days after the commencement of the attack, she was admitted into St. George's Hospital. The lower two-thirds of the thigh were greatly swollen, and pitted upon pressure. But the swelling had, notwithstanding, an elastic character, and the skin was of a glossy white. The knee was swollen also, and apparently contained fluid. The pain on attempting to move the limb, as well as upon pressure, was excessively severe. The constitutional disturbance was great. We need not mention the treatment employed. It was quite ineffectual in arresting the progress of the disease. The sufferings were very great—the swelling continued undiminished—an abscess was detected on the inferior surface of the knee, and was opened. Typhoid symptoms were established, and the patient in a short time died.

On dissection, the principal seat of the disease was found to have been in the periosteum, and cellular tissue immediately external to it. The former was stripped from the femur throughout its lower half, and lying loosened and dead in the midst of pus. The synovial capsule was ulcerated, so that the matter on the exterior of the joint, communicated with matter collected within it; the articular cartilages were partially ulcerated.

The treatment of these cases, always formidable, often fatal, is a matter of equal difficulty and importance. Active local depletion or counter-irritation, succeeded by caustic issues—perfect rest of the limb by means of splints—and calomel and opium, with colchicum internally, are the means that answer best. But the most severe cases generally prove fatal.

Mr. Mayo proceeds to describe chronic ulceration of the cartilages. We have noticed this so fully in our reviews of the work of Sir Benjamin Brodie, and of the paper of Mr. Key,* that we need not break up that ground again. We shall content ourselves with borrowing the following hints from our author.

"In studying the progress of these affections, the surgeon will remark the following points. Often when the complaint, upon being first submitted to proper treatment, has appeared to yield, and the pain and swelling have materially abated, suddenly, without any evident cause, the symptoms will return with aggravation, compelling in a few weeks some decisive step to be taken. Of all the remedies used simply, or in combination with others, rest is the most efficient. Of the others, fomentation is the next in value. Yet there is uncertainty as to the effect, even of this simple remedy: I met with an instance in which fomentation aggravated the articular pain, which was only allayed by the application of ice, that was used for a fortnight together. Leeches and caustic issues, again, which are so generally useful in these complaints, are serviceable for a period only, after which they often irritate. The articular action, when at its greatest height, and untouched by every remedy, and threatening the destruction of the joint, will occasionally spontaneously become mitigated, and rapidly diminish, and finally subside. The symptoms will sometimes be greatly aggravated by collections of turbid synovia, or pus, in the bursæ adjacent to the affected joint: the pain resulting from this cause may be removed by puncturing the cyst, and letting out the distending fluid: it is easy to ascertain, that the fluid in the adjacent bursa has no communication with the articular cavity. I have opened, with great relief to the patient, collections of turbid synovia in the sheaths of the tendons of the inner hamstring muscles, and abscesses external to the capsular membrane of the knee joint formed in the cellular tissue." 99.

We agree with Mr. Mayo that rest is a very important item in the list of remedial means. But we cannot agree with him in thinking that next to rest must be ranked fomentations. Indeed we perceive a remarkable inconsistency between his views and practice. For he goes much farther than Sir Benjamin Brodie in the opinion that the disease is inflammatory in its commencement, yet, holding such a notion, believing that ulceration of the cartilage originates in inflammation of the synovial membrane, he is still contented with remedies so inert as rest and fomentations—the very symbols of the médecine expectante.

For our own parts, we are not so satisfied as Mr. Mayo of the existence of any synovial inflammation worthy of mention, as the initiative of the disease we are considering. But we are quite satisfied, that whether that opinion be correct or not, moderately active antiphlogistic treatment is extremely useful in the early stage of the complaint. We are also satisfied that limbs and lives are not unfrequently lost—from the surgeon overlooking the early symptoms of ulceration of the cartilage, and neglecting the means we have alluded to. Whenever an individual experiences pain in an articulation, aggravated by motion and by pressure of the articulating surfaces together, that surgeon will probably sacrifice his patient who does not enjoin and ensure absolute rest, and employ local depletion and counter-irritation until either the symptoms have been removed, or it is manifest that by such means they are irremoveable. We do not pursue this subject further, as our object is not to discuss the whole treatment of ulceration of the cartilage, but merely to advert to the principle which should regulate our practice at the commencement.

---

* In the Medico-Chirurgical Transactions.

Our readers are aware that within these last few years excision of diseased joints has been loudly recommended and frequently performed by Mr. Syme, of Edinburgh. He has published many cases in which it has been successful in his hands. Of the operation Mr. Mayo expresses the following opinion, which we believe to be a close approximation to the truth.

" Of excision of the articular ends of bones in joint disease, what I have seen of it leads me to speak with no approbation. I suspect, that, where it has been performed successfully, it has been performed before the degree of exhaustion which renders an operation decidedly necessary, has arrived. When an operation is clearly required, the constitution has generally no longer force enough to support the tedious process of that restoration which follows excision. It is better, in my opinion, to wait till all chance of other cure has failed, and then to amputate. The shoulder joint appears to me the only one, in which, from the inapplicableness of amputation to the case, excision of the articular ends may be recommended." 100.

Our own observation, so far as it goes, tallies very nearly with that of Mr. Mayo. When we look at the cases detailed in the work of Mr. Syme, we are struck with the number of instances of excision of the elbow joint. Yet how seldom is amputation required for disease of that articulation, how seldom, comparatively speaking, does Nature fail to patch up, if not to cure the complaint, to produce anchylosis, if not to permit a cure. This is a staggering circumstance ; for if excision is especially applicable to cases in which amputation is very seldom necessary, it is probably of little use in those really severe cases in which the knife must be employed for the preservation of the life.

Mr. Mayo necessarily adverts to strumous caries of the bone combined with ulceration of the synovial aspect of the cartilage—to strumous caries—and to destructive disease of the articulation consequent on inflammation in contiguous parts. As an instance of the latter he mentions the following case.

*Case.* " A lad was a patient in the Middlesex Hospital, under the care of Dr. Watson and myself, for what I may describe as a suppurative fever. After cold in the head and suppuration of the ear, a return of rigor took place, and increase of fever ; and abscesses formed in succession over the great toe, over one shoulder, in the loins, and behind both hip joints. He sank under the profuse discharge. The abscesses were found to communicate with the different joints, the synovial membranes of which were in great part ulcerated away, and the cartilages of all the articular surfaces absorbed. Yet I am certain, from careful observation of the case at its commencement, that the joints were not affected in any way when the matter was first formed in their vicinity. The joints at that time contained no fluid, and were not painful, not put in pain by rubbing the articular surfaces against each other." 103.

After injuries and operations, collections of matter sometimes occur in the larger and indeed in the smaller articulations, followed, if the patient lives tolerably long, by ulceration of the cartilage on its synovial surface. We have seen great destruction of an articulation effected in this manner.

As Mr. Mayo remarks, fractures near a joint are also liable to be followed by inflammation, suppuration, and ulceration of the articular cavity. This is neither uncommon, nor unlikely, and need not detain us any longer.

The affections of the synovial membrane are reviewed in the next place by our author. He describes in order ;—1. What Sir B. Brodie has termed morbid alteration of structure of the synovial membrane ;—2. That affection in which the synovial membrane of the knee has been found covered with innumerable little processes, something like melon seeds in shape and colour, some larger, others smaller, pendulous into the cavity of the joint. Sir B. Brodie describes three preparations of this disease. One was purchased at Mr. Heaviside's collection—the second belonged to Sir C. Bell in Great Windmill-street: the symptoms in both were unknown—the third preparation, as well as the first, is in the museum of St. George's Hospital, and there is reason to believe that in this instance the excrescences were the result of long-continued inflammation of the synovial membrane. In all three the history both of symptoms and of causes is palpably defective. In Mr. Mayo's case a slight additional glimmer is afforded.

"A patient, between thirty and forty years of age, laboured under inguinal aneurism: the artery was tied, but ulcerated above the ligature. I tied the common iliac, but he sank. He had suffered for several years with occasional attacks of what was considered rheumatic gout in the knees; that is to say, with pain, swelling, and weakness. After a time, these symptoms would get better; but the knees always remained a little enlarged. They presented the appearance described above. The joints contained a few drachms of synovia." 105.

The third alteration of the synovial membrane mentioned by our author, occurred in the body of a female examined at King's College. It is a flattened tumor attached to the ligamentum mucosum, immediately below the patella; it is circular, an inch and a half in diameter, and half to two-thirds of an inch in thickness. It is covered by synovial membrane: the peduncle of the tumor is little more than a reduplicature of synovial membrane half an inch in breadth: the articular cartilages are partially ulcerated. The tumor consists of two substances; a nucleus of the size of a small bean, of a gristly or fibro-cartilaginous texture, contained in a softer membranous substance.

Mr. Mayo refers to a case related by Sir Benjamin Brodie, in which he cut into the knee-joint of a young man, for the purpose of extracting a tumor which occasioned all the symptoms commonly produced by a loose cartilage in the articulation. Violent inflammation succeeded, but the patient ultimately did well. The tumor possessed a firm fleshy structure, and was manifestly organized. It was attached to the synovial membrane below the synovial membrane, by a broad adhesion. This interesting case is related at length in the work of Sir Benjamin Brodie and is contained in our extended notice of that admirable volume.

"I have at present," says our author, "under my care a patient, in whom I suppose that a tumour of a similar description has formed upon the inner surface of the head of the tibia immediately below the edge of the cartilage. The patient is rather a nervous female, thirty-six years of age, who three months ago, having previously observed the right knee to snap on moving it, was taken with acute pain at the place of the present tumour. There was no swelling of the knee, nor pain at any other part of the joint. She was desired to apply a blister at that time, which considerably increased the pain. When the skin had healed after the blister, she first observed a tumour as big as a large pea, but flatter, on the inner surface of the head of the tibia; it was at first, and continues to be, tender on pressure. The tumour occasions her great inconvenience. She can only walk with comfort, by placing the outer edge of the foot on the ground. If she inadvertently rests upon the sole of the foot, she is seized with sickening pain in the joint, and falls down. The same pain is brought on, when the knee, being bent, is rotated inwards. The tumour is situated immediately before the internal lateral ligament; and every motion which brings the edge of that ligament a little forwarder, causes it to compress the tumour, and pain is produced. This patient derives great comfort from a bandage, which confines the motion of the joint, and prevents the ligament sliding over the tumour." 108.

In his observations upon loose cartilages in the joints, Mr. Mayo is averse to the performance of an operation, unless bandaging and means of that description have been tried without success. As Mr. M. remarks, and with justice, what renders this palliative practice the more adviseable is, that loose bodies in a joint that for several years have caused repeated attacks of pain and inflammation, occasionally, from some cause or other, cease to do so.

### Diseases of the Bursæ Mucosæ.

We see no remarks upon this head by Mr. Mayo that deserve particular attention, with the single exception of a passage in reference to enlargement of the bursa over the inferior angle of the scapula. He observes:—

"The bursa situated between the latissimus dorsi and the inferior angle of the scapula is liable to become distended with a very considerable quantity of fluid. In this state it should be punctured, and the puncture healed, if possible: the fluid is to be again let out on its reaccumulating. But the bursa, if greatly enlarged, is more likely, upon being opened, to suppurate. In the latter case, after discharging for some weeks, it will probably dry up, and the case terminate favourably." 111.

We notice this passage in order to enjoin caution in meddling with this, or indeed with other bursæ, when considerably enlarged. We have seen death occur from the suppuration induced in this bursa by a puncture. We have also seen death ensue from the employment of the seton. The symptoms in both instances were of a typhoid character—such symptoms as occasionally follow the exposure of the cavities of large abscesses. On the whole we are not by any means enamoured of operations on the bursæ, unless, as in the instances of the bursa above the patella and the olecranon, they are purely subcutaneous.

Mr. Mayo proceeds to the pathology of the muscles, after which he takes up that of the cellular tissue, the nervous system and the skin. The consideration of the morbid alterations of these tissues we are under the necessity of deferring till our next number. The rapid advances of pathology render an accurate acquaintance with its facts indispensable to every one who aspires to even a moderate amount of professional attainments. On this account we have for some time past endeavoured to present in each number of this Journal, an article devoted to the exposition of some pathological points. We are anxious to avoid deferring a notice of the last fasciculus of Dr. Carswell, and to include with it and with the review we are now quitting some portion of the later labours of Cruveilhier. We must therefore, as we said before postpone till our next number an analysis of the remainder of the first (the present) Part of Mr. Mayo's Outlines of Pathology. Yet before we quit it, although but for a season, we would recommend the many purchasers of the Physiology of the same author, to make themselves possessors of this kindred system of Pathology. The two go hand in hand, and—

Each gives to each a double charm
Like pearls upon an Ethiop's arm.

The eighth Fasciculus of Dr. Carswell's Illustrations of Pathological Anatomy lies before us. It is occupied with the consideration of the morbid product—Pus.

Dr. Carswell starts with an account of the various theories of the mode of production of pus, which preceded the present, and, no doubt the true belief, that it arises from secretion. We need not resuscitate the absurdities that prevailed before the advent of the new doctrine, but we think the short sketch presented by our author of the manner and the period at which that doctrine was introduced, may not be without interest to our readers. We shall transcribe the passage to which we refer.

"The opinion, however, that pus is formed in the capillary vessels by a process analogous to secretion, was first suggested by Dr. Simpson, of St. Andrew's, in the year 1722. 'If any foreign body,' he observes, 'be introduced between the edges of a wound, and the external air excluded, pus will continue to be discharged as long as you please; so that by this means, a *kind of new gland* is, as it were, produced. But if the wound be irritated or too much compressed, the properties of the liquor discharged will be immediately altered, as is well known to surgeons. Hence it follows that nothing is more easy than to change the secretions and humours of the body without the addition of any new matter or ferment, merely by changing the diameters and number of the secreting vessels.' De Haen announced a similar opinion about the year 1756, from having observed that pus is often expectorated for a great length of time, by patients affected with phthisis, in whom, after death, no mark of ulceration could be perceived, not even the place in which the pus had been formed. From these circumstances, De Haen was led to believe that pus must have been immediately secreted from the blood, and adds, 'that although the blood appears to be a homogeneous fluid, yet it is manifest that there is something in it when collected which is of a tenacious consistence, of a whitish, or a yellowish colour, and which might be called the matter of pus.' The opinions contained in the writings of De Haen seem to have suggested to Dr. Morgan the new theory of the formation of pus, which, as has been remarked by Professor John Thomson, was first embodied into a general doctrine, and subjected to a full discussion in his inaugural dissertation printed at Edinburgh in the year 1763, entitled, Puopoieses, sive Tentamen Medicum de Puris Confectione. The principles of the new theory of the formation of pus which had been thus introduced, soon found several able supporters, among whom Mr. Hunter may be regarded as the first who furnished satisfactory evidence of their accuracy, by means of new facts and illustrations derived from his experiments on living animals, and his researches on suppurative inflammation and on pus." 2.

Dr. Carswell presents a view of the opinions of Mr. Hunter on the subject of pus and of suppuration, and shews that that indefatigable physiologist considered both a consequence of inflammation, and the former a remove further from the nature of the blood than the lymph poured out in the adhesive inflammation. This sentiment has been confirmed by the recent investigations of Gendrin, who observes that between the purulent fluid of inflamed tissues and organized coagulable lymph, there is only a difference of degree.

Dr. Carswell next offers a view of the process of suppuration, as it occurs in parts in which its phenomena can be distinctly seen, and their nature can be, in some degree at least, determined.

Gendrin, who investigated, under a great variety of circumstances, the changes that take place in inflamed tissues, gives a minute account of the process of suppuration. He excited inflammation in the web of the frog's foot and in the mesentery by means of boiling water, the actual cautery and the seton, and followed with the microscope the gradual development of pus from the globules of the blood. He relates as having distinctly seen the globules of the blood, after having been brought to a state of stagnation in the capillaries, becoming deprived of their colouring envelope and opaque, and assuming a yellowish grey colour approaching to that of pus; that he has then traced them moving slowly in the capillaries or in new-formed vessels, and as they advanced towards the edge of the ulcer or eschar occasioned by the violence of the inflammation, gradually acquiring all the physical characters of perfect pus. Kaltenbrunner, than whom there does not appear to have been a more able, cautious, and philosophical observer, had, before the researches of Gendrin were published, arrived at nearly the same results. The microscopical researches of this author, however, would seem to shew that not only is the blood which is carried into the inflamed tissue, but likewise a portion of the solids, converted into pus; for he states that small granular bodies are seen to separate from the *parenchyma*, to pass into canals which are formed for their reception, and to mingle with others of a similar kind coming from the blood, both of which are gradually converted into true granules of pus. The result of these researches is, that the formation of pus is a consequence of a modification of the blood, manifested especially by a change in the colour, transparency, and bulk of this fluid, after its circulation has been arrested in the capillaries by inflammation—that this change in the globules takes place in the capillary vessels, and that these vessels conduct the globules in this state to the exterior, where they appear combined with the serum of the blood under a peculiar liquid form, or that which we call pus.

" Such," says Dr. Carswell, "is one mode in which pus is formed, that in fact, which has been likened to the process of secretion. There is, however, another mode, which, to a greater or less extent, is, perhaps, always in operation at the same time with the former, but which, under certain circumstances, is effected alone, and from its not having been properly understood has created much confusion, and given rise to opinions the most repugnant to the principles of a sound and philosophical pathology. I allude to the formation of pus in the blood, under circumstances in which the influence of the capillary system, as exercising a function of secretion, can have no part. Some evidence has already been adduced in favour of this mode of formation of pus, and which may be called *extra-vascular*. Thus we have seen, that besides the conversion of the globules of the blood, contained in the capillaries, into pus, there was also observed a similar change in those of the blood effused into the cellular tissue around these vessels; in fact, the whole of the blood, *intra* and *extra*-vascular, was seen to undergo the same gradual change of colour from red to yellowish grey; the globules in both situations became opaque, acquired an increase of bulk, and passed slowly towards the surface of the part, either in the original or new-formed capillary vessels in the form of pus." 5.

Our author proceeds to instance what happens to the effused blood in violent inflammation of the cellular tissue. That tissue, with its capillaries, minute arteries, and veins, is distended with that fluid. When suppuration supervenes, the gradual discoloration, softening, and conversion of the whole into pus, succeed to each other, and must be produced on the same principles as those observed in the blood of a single capillary under the microscope, for the pus is equally perfect in both cases. Dr. C. has on several occasions been able to satisfy himself that this mode of formation of pus often occurs to a considerable extent in blood effused into the cellular tissue, from external violence, and when followed by acute inflammation; but he has most frequently observed it in the blood which has ceased to circulate in inflamed veins. Thus, in the first case, the conversion of the blood into pus is proved by this latter fluid alone being found some time after in the swelling which had been originally produced by the effused blood. Gendrin has put this fact beyond all doubt by direct experiment. "If, after having injected," observes this author, "a great quantity of blood into the subcutaneous cellular tissue, a seton is passed through the same tissue in order to excite a certain degree of inflammation, the blood is rapidly converted into pus, as if it had escaped from the vessels themselves of the part."

"In phlebitis again," continues our author, "the conversion of the blood into pus can often be most satisfactorily demonstrated. As soon as inflammation of a vein has acquired a certain degree of severity, one of the most remarkable circumstances which attracts our notice, is the cord-like hardness of the vessel. The blood has ceased to circulate through it—it has coagulated. At a subsequent period, however, we find that this state of the vessel becomes less conspicuous, and disappears, sometimes suddenly, no cord-like swelling or induration being afterwards felt. Now, further examination shews that the latter occurrence was the result of the blood having been converted into pus." 5.

The gradual phases of this conversion are exhibited by the dissection of a phlebitic limb. In the veins which are but slightly inflamed, the blood is found in a state of fluidity, and presents the colour of venous blood; in those in which the inflammation is marked by great vascularity, serous and albuminous effusion in the middle and external coats, it is coagulated, slightly adherent to the internal membrane, or united to it by a thin layer of unorganized coagulable lymph, and exhibits the colour of arterial blood. As we approach those portions of the veins in which the inflammation commenced, or in which it has proceeded to the suppurative stage, the arterial colour of the blood passes gradually into a pale yellow or that of fibrine, of which it has at first also the consistence, but becoming soft, pulpy, and creamy, until it is at last lost in the puriform matter contained in the vessels or in the surrounding tissues.

We can bear sufficient testimony to the accuracy of these statements. It was but the other day we examined the condition of the veins in a case of inflammation of the femoral and iliac veins. The coagulum was converted in parts into a substance so exactly similar to pus, that independently of refined analysis there was no appreciable distinction between them.

"From all these facts," continues Dr. Carswell, "it must be evident that the capillaries could have no share in the purulent transformation of the blood in the examples which have been brought forward of this change, and therefore it follows that the formation of pus cannot be restricted to a morbid process induced in these vessels by inflammation, and the subsequent separation of the elements of this fluid from the blood. In these experiments and observations there are, however, three striking circumstances which, from their constancy, the regularity of their occurrence, and the uniformity of their results, must be regarded as having a common origin, or as consequences the one of the other: these are, the cessation of the circulation, the coagulation of the blood, and the conversion of the fibrine or globular structure of this fluid into pus.

These three series of changes we have always seen succeeding to each other, that is to say, the globules of pus made their appearance in the fibrine of the blood which had become deprived of its colouring matter after having been brought to a state of stagnation in the capillaries, in arterial and venous trunks, and in lacerated excavations formed in the cellular tissue. But we have also seen that these changes were always preceded by inflammation of the tissue in which the blood was contained, and the pus that was immediately derived from this fluid. That inflammation was the common origin of these changes cannot, therefore, admit of a doubt; and as it has been proved that they constitute the process of suppuration, the legitimate conclusion at which we arrive is, that this morbid process is essentially dependent upon inflammation as its efficient cause." 6.

We are not quite sure that we have made much advance in our real knowledge of the mode in which the process of suppuration takes place. It is generally admitted that pus is a secretion from the capillaries, and, being a secretion from those vessels, it must of course be formed from the blood. Supposing the preceding observations to be accurate, they carry us only one short step higher, when they prove the blood whence the pus proceeds must previously be stagnant. That mere stagnation of blood is insufficient, is proved by the large extravasations that are quietly although very slowly absorbed. Inflammation, then, must be added to the stagnation of the blood, and this much disputed process must be called into the field at last. There are two circumstances that ought not to be lost sight of;—1, that in certain cases of stagnation and coagulation of the blood, where inflammation is not supposed to exist, softening in the centre of the clot takes place. This we have seen occur in the "polypi" of the heart, and in the museum of St. George's Hospital is a drawing exhibiting an instance of this description. 2, A surface continues to suppurate for a length of time. Is it meant that the blood must still be stagnant in the capillaries, in order that the quantities of pus which we see a wound secrete from day to day, may be formed and dissolved, as it were, from that coagulum? These considerations induce us to extend some degree of doubt to the theory or the observations which would make us presuppose coagulation of the blood as a necessary antecedent to suppuration.

Our author adverts next to a very interesting subject, the nature and the mode of formation of what have been denominated the Purulent Deposits.

Dr. Carswell commences the consideration of the origin of these deposits by a rather startling announcement. He observes that, as the deposits in question occur in various parts of the body without being accompanied by the usual characters of inflammation, the point has been mooted—is the presence of pus always to be considered as a product of inflammation of the part in which it is found? He conceives that much of the difficulty attending the solution of this question would be done away with if the distinction between suppuration as a vital act, and the mere presence of pus as its product, were previously well understood. If, he proceeds, pus is found in an organ where none of the characters of inflammation can be detected, the necessity of the above-mentioned distinction must be obvious. As an instance of the formation of pus without appreciable marks of inflammation he cites the fact of pus being found in the centre of cardiac polypi. But it is his mode of using this fact, which, as we observed just now, rather startles us.

"I have never (says our author) met with a case of anomalous formation of pus, either in the cavities of the heart, in the cellular or parenchymatous structure of organs, or in the cavities of serous membranes, without finding at the same time inflammation and suppuration to a greater or less extent in some remote organ. It may, indeed, be asserted, that this is also the almost uniform result of the researches of other pathologists, although Andral, Maréchal, and some others, have rather vaguely stated that they have met with puriform matter in fibrinous concretions of the heart, without the presence of pus being detected in any other organ of the body. Such cases, several of which I have also seen, are very different from those now under consideration. The fluid matter of these concretions, so far as my observation goes, never resembles pure pus; it is a thin, grumous, grey, or reddish-coloured fluid, but probably puriform in its nature, as it resembles the contents of those concretions which are formed during life in the cavities of the heart, in some cases of inflammation of the internal membrane of this organ, succeeding to rheumatism of the joints. If such be the origin of puriform collections of this kind, they of course come under the head of suppuration, and do not form an exception to the law, that the formation of pus in the blood and in the other parts of the body which I have enumerated, under circumstances which disprove its connexion with inflammation as cause and effect, has never been shewn to take place without being preceded by suppuration in a remote organ." 8.

Now on this point we are disposed to join issue with the Doctor. We have already referred to a drawing of pus in the centre of cardiac polypi, contained in the museum of St. George's Hospital. We saw the case from which that drawing was derived. The patient laboured under passive dilatation of the heart and dropsy. He had no perceptible suppuration in any other organ, yet the pus in the cardiac coagula was as like pus as any we have ever seen. The evidence of its being pus was quite as good as is generally obtained and admitted. At the same time, we are disposed to agree with Dr. Carswell that pus, as a rule, is either the result of inflammation of the part, or of suppuration (the consequence of inflammation), in other parts.

Our readers may know that the cause of the purulent deposits has been, of late years, a vexata quæstio. Some have placed it in nervous agency—some in the necessary antecedence of phlebitis. We cannot coincide with those who see in phlebitis the whole and sole cause of these secondary deposits; but still we believe that it is a very common one—more common than we were formerly led to suppose. The following extract embodies an epitome of many of the facts connected with the origin of these formations.

"The situation of the pus in these cases of suppurative inflammation is another circumstance which deserves particular notice. In the great majority of cases, this morbid product is found in the veins of the affected part. In idiopathic phlebitis, in phlebitis of the uterus following delivery, and even in phlebitis after bloodletting, it is sometimes found only in the veins; whereas, in phlebitis succeeding to external injuries or operations, it exists, often extensively, at the same time, in the intermuscular cellular tissue, in the canals and cancelli of the bones. It is found in the smallest veins that can be traced by dissection, in the largest trunks of the extremites, and pelvic viscera, and even in the vena cava. In some cases, the quantity of pus does not amount to a drachm, in others it measures several ounces. The number of veins in which it is found varies from a very few, small or large, to the whole of those of the arm or forearm, or of the uterus. The arteries, in such cases, never contain any pus, nor have I met with it to any extent in the lymphatics, except in uterine phlebitis, in some cases of which these vessels were distended with it, the veins being in a similar state, or containing only a very small quantity." 8.

On the whole, Dr. Carswell is led to conclude that the purulent deposits result from the entrance of pus into the circulation—a theory which, though defective, is probably nearer the truth than any other that has yet been advanced. Dr. Carswell thinks it both satisfactory and complete. The latter we do not think it, as certainly the secondary inflammations occur, where no primary suppuration elsewhere existed. The steward of St. George's Hospital was detected in the commission of a grave offence. The disclosure affected his mind to a very extreme degree. He fell into a state of low fever, and in a few days died. His lungs were found studded with the purulent deposites, while all the rest of the body was sound. We have dwelt on this subject so fully on previous occasions, that we shall not go into the discussion again. One other point, and we have done.

"In order to complete the history of purulent deposits, it may be remarked that the number of organs in which they occur, varies considerably. In some cases only one organ is affected, sometimes to a very small, sometimes to a great extent; whilst in other cases a greater or less number of organs are affected in a similar manner. The lungs and liver are far more frequently affected with these deposits than any of the other organs of the body, whatever may be the seat of the lesion in which they originate. Recent observations shew that those of the liver are not more frequent after injuries of the head than of the extremities. Of all the organs of the body, the kidneys are least frequently the seat of purulent deposits. The few cases which are recorded as examples of this kind, appear to have been the result of inflammation, extending to the kidneys from neighbouring organs, and succeeding to the operation of lithotomy, to injuries of the spine, to the presence of calculi, and to various diseases of the pelvic viscera. We cannot, perhaps, appreciate the importance of this circumstance, but it is extremely probable that it is to be accounted for by the separation of the *material* cause of these depositions from the blood carried into these organs, and its excretion along with the urine." 11.

We agree with Dr. Carswell, that the kidneys are very seldom affected; but we have seen them so twice or thrice. In the St. George's School of Anatomy, in Kinnerton Street, there is a preparation, exhibiting distinct purulent deposit in the kidney, unaccompanied with any contiguous inflammation.

The fasciculus before us terminates with some general observations on the properties of pus.

Dr. Carswell remarks, and remarks with justice, that the attempts to obtain accurate discriminating marks between pus and mucus have been ineffectual, and, if effectual, would be practically useless. For as mucous, and indeed serous membranes, when inflamed, furnish pus, the latter is no certain test of ulceration. The physical characters of pus are familiar, and we need not recapitulate them here. Dr. Carswell's account of its chemical properties is succinct and good.

He remarks that, being a product of secretion, pus must be chemically modified by the nature of the tissue from which it is derived, as well as by those various morbid conditions which are known to modify the products of secretion in general. It is on this principle, continues Dr. C. that we explain the difference which exists, particularly at the commencement and termination of suppuration, between the pus furnished by serous and mucous membranes, the quantity of albuminous matter being much greater in the former than in the latter case; and, as has been observed by Gendrin, that the pus furnished by the granulations in caries contains a greater quantity of the phosphate and muriate of lime; the puriform discharge of scrofulous ulcers a larger proportion of soda and the muriate of soda; and that which is found in the tissues surrounding the joints in gout, an excess of the carbonates, phosphates, and, perhaps, the urate of lime. A difference in the degree of the inflammatory excitement of the affected part; the supervention of an acute disease in a remote organ; a sudden emotion, whether followed by great excitement or depression, &c. produce also obvious changes in the physical, if not in the chemical characters of pus.

Dr. Carswell notices Mr. Pearson's arrangement of the varieties of pus:—1, creamy pus; 2, curdy pus; 3, serous pus; 4, slimy pus; to which Andral has added a 5th, concrete pus, which is the same as the curdy of Mr. Pearson, but deprived of a great part of its serum by absorption, such as it is often met with in scrofulous abscesses. They are found to be composed of albumen in a state of concretion, water, extractive matter, a substance resembling adipocire, and various salts. Chemical analysis, therefore, does not establish any important distinction between pus and the serum of the blood, the difference, in this respect, between the two fluids consisting in the state of concretion of the albumen, and a modification of the extractive matter.

In conclusion, our author observes that investigation has not yet discovered in what the specific properties of pus consist. The matter of a syphylitic sore, a small-pox pustule, and a common phlegmonous abscess, does not appear to display peculiar properties in those various states and circumstances.

The plates of this fasciculus are, as usual, beautiful. They represent the purulent deposits in their various forms and their various situations. Phlebitis, too, is shewn, and the whole are well worthy of purchase and of study. As a work, these Illustrations of the Elementary Forms of Disease must be deemed possessed of high interest and merit, and that medical library is incomplete, that medical man's store of useful volumes is imperfect, which does not contain this among its biblical treasures.

